# The rare case of oesophago-pericardial fistula secondary to pulmonary tuberculosis

**DOI:** 10.1093/jscr/rjac422

**Published:** 2022-09-24

**Authors:** Kavina Kaur Sidhu, Doruk Seyfi, Ngee Soon Lau, David Andrew Yeo

**Affiliations:** Department of Upper GI Surgery, Royal Prince Alfred Hospital, Camperdown NSW, Australia; Department of Upper GI Surgery, Royal Prince Alfred Hospital, Camperdown NSW, Australia; Department of Upper GI Surgery, Royal Prince Alfred Hospital, Camperdown NSW, Australia; Department of Upper GI Surgery, Royal Prince Alfred Hospital, Camperdown NSW, Australia

## Abstract

We report the case of a healthy 35-year-old male with two rare pathologies: pneumopericardium and oesophago-pericardial fistula (OPF) secondary to tuberculosis. Purulent pericarditis and cardiac tamponade are known complications with potential for significant morbidity and mortality. Unfortunately, the symptoms of OPF are non-specific often delaying diagnosis. There is no gold standard for treatment or determinant of when nonsurgical versus surgical approach should be considered. Anti-tuberculous therapy alone is often adequate however an oesophageal stent was utilized in this case to rapidly gain control of the fistula and prevent ongoing contamination from mediastinitis.

## INTRODUCTION

Tuberculosis (TB) infects ~33% of the world’s population and kills ~1.5 million people annually [1]. It is the world’s leading cause of death due to a single infectious cause [2]. The key risk factor is travel to or residence in a high-risk country.

Oesophageal TB is rare and usually occurs in immunocompromised patients. Breach of the pericardium due to the close anatomical proximity of the oesophagus and pericardium occurs with direct communication between the pericardial sac and oesophagus (air containing viscus) resulting in an oesophago-pericardial fistula (OPF). Causes of OPF can be benign or malignant. In non-malignant OPF, TB should be considered especially in the immunocompromised and those from high TB prevalence regions. OPF may be managed conservatively with anti-TB treatment, however recently, endoscopic stenting is a feasible option.

This is an interesting case of a healthy 35-year-old male with pneumopericardium and OPF secondary to TB.

## CASE REPORT

A 35-year-old labourer presented with a 1-month history of non-productive cough and lethargy. He immigrated to Australia from India in 2015 with normal pre-migration health checks and last visited India 3 years previously. He was a current smoker, but otherwise well.

The patient was pale, tachycardic and febrile but not in respiratory distress. His jugular venous pressure was elevated without palpable lymphadenopathy. He had elevated inflammatory markers (white cell count, WCC 14.2 × 10^9^/l; C-reactive protein, CRP 143 mg/l), an acute kidney injury (creatinine 133 umol/l, eGFR 59 ml/min/1.73m^2^) and was anaemic (Hb 81 g/l).

**Figure 1:**
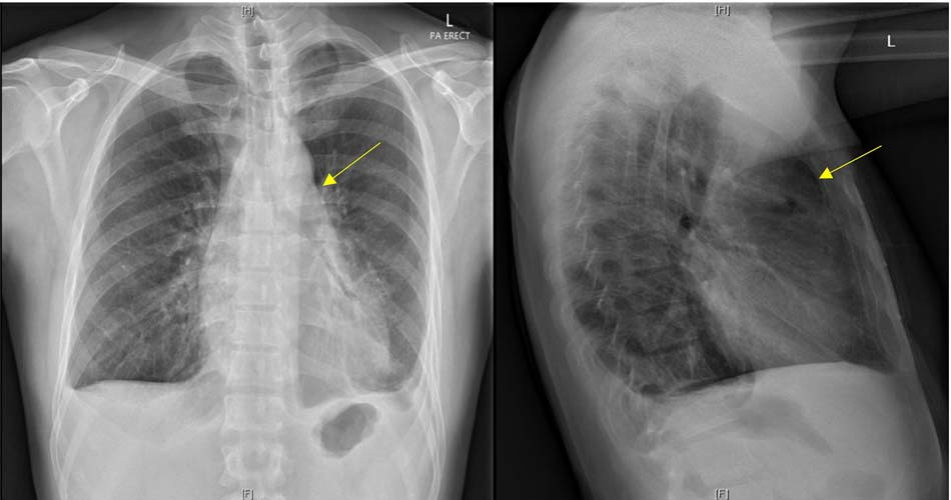
Postero-anterior erect and lateral chest radiograph. Lucency over the left heart border and anterior middle mediastinum consistent with a pneumopericardium.

**Figure 2:**
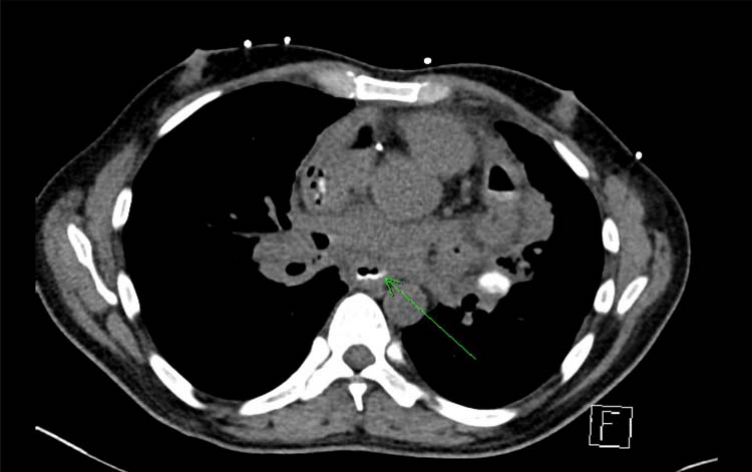
CT with water-soluble oral contrast. Trace amount of contrast that extends to the left lateral aspect of the oesophagus representing a thin tract.

Initial chest X-ray demonstrated lucency over the left heart border and anterior mediastinum suggesting pneumopericardium (Fig. 1). A computed tomography (CT) scan of the chest demonstrated multiple pericardial collections containing loculated gas and fluid with calcification causing stenosis of the superior vena cava. An OPF was suspected (Fig. 2). Bilateral tree-in-bud nodularity reflecting active pulmonary infection with diffuse bronchitis and endobronchial plugging were also present.

Pulmonary TB was confirmed by sputum polymerase chain reaction. Empiric anti-TB treatment and broad-spectrum antibiotics for mediastinitis were commenced.

**Figure 3:**
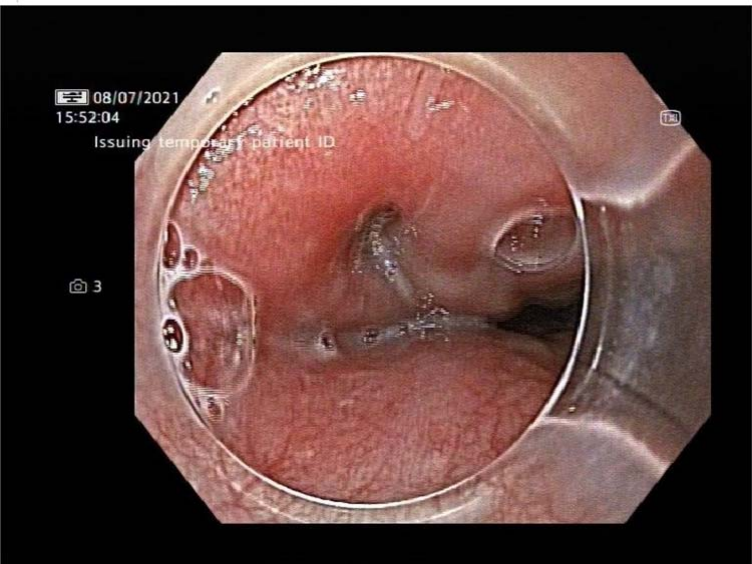
Upper gastrointestinal endoscopy demonstrated three mucosal defects 31 cm from the incisors.

An OPF was confirmed on chest CT with water-soluble oral contrast. Endoscopy demonstrated three mucosal defects 31 cm from the incisors (Fig. 3). A fully-covered WallFlex stent (23 × 15 mm, Boston Scientific) was positioned to cover the OPF. The stent was secured proximally to the oesophageal mucosa using resolution clips and distally to the stomach using a 3–0 PDS trans-gastric suture placed laparoscopically. The patient improved and it was decided not to proceed with pericardial drainage.

Fluoroscopic swallow on Day 6 post-operatively confirmed no extravasation of contrast and satisfactory stent position. He was discharged a week later on anti-TB treatment and Moxifloxacin for 8 weeks.

Chest X-ray 4 weeks post-procedure demonstrated stent migration into the stomach. It was removed endoscopically without complication.

## DISCUSSION

OPF is rare with 83% in-hospital mortality [3]. Forty-nine cases are documented worldwide diagnosed through radiographic studies, surgery or autopsy [4]. It occurs secondary to local extension of infection or TB reactivation. OPF causes are benign or malignant—most commonly due to ulceration and foreign body. TB should be considered in the immunocompromised and those from high TB prevalence regions. Tuberculous pericarditis develops in 1–2% of pulmonary TB cases [5]. Purulent pericarditis and cardiac tamponade are complications with potential for significant morbidity and mortality.

The symptoms of OPF are non-specific, delaying diagnosis. Oesophageal fluoroscopy with water-soluble contrast is the best diagnostic study but CT is the most complete method of examining the mediastinum [6]. Biopsies, cultures and staining must be performed. An echocardiogram is essential in pericardial assessment.

There is no gold standard for treatment or determinant of when to consider nonsurgical versus surgical approach. OPF management consists of anti-TB treatment and recently endoscopic stenting has supplanted oesophagectomy and cervical oesophagostomy [7].

Oesophageal stents were approved for use in 1845 [7] with self-expanding metal stents (SEMS) providing great flexibility and radial force to maintain patency and position with easier insertion and less migration. A fully-covered stent is effective to seal up the fistula entrance and control mediastinal sepsis. This minimally invasive approach proved effective and serves as definitive treatment especially in cases where surgical treatment was once considered only treatment of choice e.g. OPF post AF catheter ablation without atrial involvement [8]. SEMS is the standard of care in unresectable malignant oesophageal obstruction and oesophageal-respiratory fistulas. Emerging roles in benign conditions such as anastomotic leaks or perforations are promising [9]. Stent migration is the most common complication with a frequency of ~75% [10]. Our approach to fix the stent proximally and distally to reduce the risk of migration has never been previously described.

The role of surgery for TB-related OPF remains controversial. All OPF secondary to TB did not require surgical intervention except in one paediatric patient [11]. Surgery is highly invasive, reserved for recurrent oesophageal fistulas or failure of medical management but the evidence is limited only to case reports. Therefore, other options are typically pursued first.

The rarity of TB-related OPF and conservative management approach makes this case unique. Stents are useful and relatively non-invasive to control sepsis without need for major surgery (oesophagectomy, sternotomy, pericardial debridement etc.). Stents should be considered a good option for sepsis control prior to invasive surgery in combination with anti-TB treatment especially in clinically stable patients.

## Supplementary Material

Editted_Gastroscopy_rjac422

## References

[1] White Z , Painter J, Douglas P, Abubakar I, Njoo H, Archibald C, et al. Immigrant arrival and tuberculosis among large immigrant- and refugee-receiving countries, 2005-2009. Tuberc Res Treat 2017;2017:1–8. 10.1155/2017/8567893 PMC5382300 28424748

[2] Trauer JM , Williams B, Laemmle-Ruff I, Horyniak D, Caplice LVS, McBryde ES, et al. Tuberculosis in migrants to Australia: outcomes of a national screening program. Lancet Reg Health West Pac 2021;10:100135. 34327348 10.1016/j.lanwpc.2021.100135 PMC8315463

[3] Al-Ajmi J , Al-Soub H, El-Deeb Y. Pyopneumopericardium due to esophago-pericardial fistula in patient with tuberculous pericarditis. Saudi Med J 2007;28:969–70. 17530123

[4] Cyrlak D , Cohen AJ, Dana ER. Esophagopericardial fistula: causes and radiographic features. AJR Am J Roentgenol 1983;141:177–9. 6602516 10.2214/ajr.141.1.177

[5] Jurado LF , Pinzón B, De La Rosa ZR, Mejía M, Palacios DM. Tuberculous pericarditis. Biomedica 2020;40:23–5. 32463604 10.7705/biomedica.4911 PMC7449104

[6] Rämö OJ, Salo JA, Isolauri J, Luostarinen M, Mattila SP . Tuberculous fistula of the esophagus. Ann Thorac Surg. 1996;62:1030–2. 10.1016/0003-4975(96)00471-7 8823085

[7] Wang Y , Liou DZ, Tsai EB, Lui NS. Treatment of a benign esophagopericardial fistula with an esophageal stent—a case report. Ann Esophagus 2020;3:30–0.

[8] Deneke T , Sonne K, Ene E, Berkovitz A, Nentwich K. Esophagopericardial fistula: the wolf in sheep’s clothing. JACC Case Rep 2021;3:1136–8. 34471898 10.1016/j.jaccas.2021.04.043 PMC8314118

[9] Chan BY , Chan CK. Retrievable endoscopic stenting for tuberculous oesophagopleural fistula with empyema. Hong Kong Med J 2017;23:89–92. 28184018 10.12809/hkmj154670

[10] Farkas ZC , Pal S, Jolly GP, Lim MMD, Malik A, Malekan R. Esophagopericardial fistula and pneumopericardium from caustic ingestion and esophageal stent. Ann Thorac Surg 2019;107:e207–8. 30179624 10.1016/j.athoracsur.2018.06.087

[11] Ko Y , Lee H-Y, Lee Y-S, Kim M-Y, Lee Y-M, Seon Kang M, et al. Esophagomediastinal fistula secondary to multidrug-resistant tuberculous mediastinal lymphadenitis. Intern Med 2014;53:1819–24. 25130118 10.2169/internalmedicine.53.2145

